# Strengths and challenges faced on the implementation of global surgery education courses: a scoping review

**DOI:** 10.1590/acb416426

**Published:** 2026-11-06

**Authors:** Sarah Lopes Salomão, Beatriz Laus Pereira Lima, Laynara Vitória da Silva Vieira, Ana Maria Bicudo Diniz, Ayla Gerk Rangel, Luiza Telles, Lauren Kratky, Nivaldo Alonso, Cristiano Tonello, David Mooney, Cristina Pires Camargo

**Affiliations:** 1 Universidade de São Paulo – Programa de Pós-Graduação da Faculdade de Medicina de Bauru – São Paulo (SP) – Brazil.; 2 Universidade São Francisco – Faculdade de Medicina – Bragança Paulista (SP) – Brazil.; 3 Universidade Federal do Piauí – Faculdade de Medicina – Teresina (PI) – Brazil.; 4 Universidade de São Paulo – Faculdade de Medicina – Ribeirão Preto (SP) – Brazil.; 5 McGill University – Faculty of Medicine and Health Sciences – Montreal (QC) – Canada.; 6 Universidade de São Paulo – Faculdade de Medicina – Programa de Pós-Graduação em Cirurgia Plástica – São Paulo (SP) – Brazil.; 7 Johns Hopkins University – School of Public Health – Baltimore (MD) – United States.; 8 Harvard University – Harvard Medical School – Program in Global Surgery and Social Change – Cambridge (MA) – United States.; 9 Universidade de São Paulo – Faculdade de Medicina – São Paulo (SP) – Brazil.; 10 Universidade de São Paulo – Faculdade de Medicina de Bauru – Bauru (SP), Brazil.; 11 Harvard University – Harvard Medical School – Boston Children’s Hospital – Boston (MA), United States.

**Keywords:** Global Health, Education, Medical, Academia, Developing Countries

## Abstract

**Purpose:**

To evaluate educational initiatives in global surgery by answering: what are the characteristics, educational strategies, and reported outcomes of formal global surgery courses and training initiatives for healthcare professionals and medical students worldwide?

**Methods:**

All available global surgery educational initiatives reported in studies, with no specific timeframe restriction, were included. We analyzed general information about the study and the global surgery courses reported.

**Results:**

The search strategy retrieved 1,040 records, 250 of which met the criteria for full-text review, and 119 which were included in the final analysis. Among the studies, 68.9% (n = 82/119) of them that underwent full-text review had first authors affiliated with institutions in the United States of America. Nevertheless, most courses took place in low and middle-income countries (50.42%—n = 60/119), and 46.28% (n = 56/119) of the courses corresponded to general medical education. Most courses were both practical and theoretical (32.77%—n = 39/119). The courses targeted mainly local surgical residents (27.15%—n = 41/119).

**Conclusion:**

The field of global surgery has been expanding since before 2015, but nearly 10 years later, it lacks a standardized educational framework. Often, high-income country authors lead practical courses in low and middle-income countries, underscoring the need for local perspectives.

## Introduction

In 2015, the Lancet Commission on Global Surgery (LCoGS) formally established the field of global surgery to address disparities in surgical, anesthesia, and obstetric care^1^. Since then, global surgery has emerged as a critical component of global health initiatives, aiming to improve surgical care access and delivery across different regions and populations worldwide^1^. In this context, LCoGS consistently highlights education, training, and capacity building as a crucial element for achieving surgical welfare across health systems^1^.

Education as an essential pillar of global surgery has the potential to improve surgical care in low- and middle-income countries (LMICs), promote health equity, and empower local professionals^2^. Previous studies mention the importance of education and training of surgical practitioners and allied healthcare workers^3^. Education refers to academic theoretical learning and knowledge, while training means practical clinical skill development^3^. Integrating global surgery into educational programs is essential for cultivating a generation of healthcare professionals who are both conscious of global health disparities and better prepared to address them^4^. Therefore, the landscape of global surgery education and training initiatives benefits from being further explored.

Our study unites the voices of previous efforts, calling for a reassessment of global surgery education and training. As the LCoGS marks its 10th anniversary in 2025, sustaining efforts to advance educational and training initiatives is essential. To this end, this scoping review explored both surgical education within global surgery and global surgery education as a distinct field, offering a comprehensive assessment of the current global surgery education landscape. We considered both practical and theoretical courses, having the opportunity to study training courses, but also global surgery as academic content. No specific timeframe was applied to the search, as some educational initiatives in global surgery predated the publication of the LCoGS. Therefore, restricting the search period could have resulted in the exclusion of relevant earlier initiatives and limited a comprehensive assessment of the field’s development.

## Methods

We conducted a scoping review of the literature to investigate the current state of global surgery education and training. This study followed the Preferred Reporting Items for Scoping Reviews and Meta-Analyses (PRISMA) guidelines and checklist for conducting scoping reviews. This review was registered with the National Institute for Health Research as PROSPERO 2024 CRD42024541692 and has its protocol available on the link https://www.crd.york.ac.uk/PROSPERO/view/541692. This review searched articles published before December 4th, 2023.

Inclusion criteria included papers that discussed global surgery courses and/or workshops (theoretical courses or practical training in surgery aimed at global surgery actions), targeted any career level (undergraduate, graduate, and/or post-graduate students), and included information about the curriculum or insights about global surgery education. This study considered all surgical specialties, countries, languages and years of publication.

We excluded survey studies aiming to understand the interests of students or professionals in global surgery. However, surveys evaluating the quality or satisfaction related to completed courses were included.

### Search strategy

The search strategy integrated two groups. The first group had topics related to medical education, and the second one with the keyword “global surgery.” The strategy was adapted and applied to the following databases: Scopus, PubMed, Embase, Virtual Health Library, and Web of Science.

### Selection and data collection processes

To structure each phase (screening, full-text analysis, and data extraction), we used Covidence Better systematic review management. In the first screening phase, two reviewers (SLS; LT) independently screened each record by title and abstract. Disagreements were solved with a third reviewer (CPC). The second phase of the selection process (full-text analysis) had a group of four independent reviewers (SLS; BLPL; LVSV; LT). The disagreements from full-text analysis were solved by a fifth reviewer (CPC). No assumptions were made about any missing or unclear information from the studies. When the study did not present an outcome for extraction, it was filled as no data available (NDA).

We collected data on the landscape of global surgery courses, covering their main topics, target audience, duration, geographic distribution, organization location, and project execution. Quantitative reach, student satisfaction with learning, and assessing equity in participation by students from high-income countries (HICs) and LMICs were also evaluated. This encompassed both theoretical and practical aspects of the courses.

### Statistical analysis

We described the data according to the nature and distribution of the variables. To measure the association of global surgery education, we performed a median quantile regression analysis to explore the association of course, study, country-level, and the number of global surgery students relative to the physician workforce in the corresponding country. The number of global surgery students and physicians was standardized to the population size of each country before calculating the student-to-physician ratio. R-squared (R^2^) was used to measure the proportion of association. The threshold of the *p*-value was set at < 0.05. We collected the geographical data from the World Bank Open Data, available on: https://data.worldbank.org/country. Tabulation and analysis were performed with Microsoft Excel 365 (Microsoft Excel: Office 365, Redmond (WA), United States of America). STATA v18 was used for our analysis (Stata Statistical Software: Release 18, United States of America).

Given the non-parametric nature of the data, a quantile regression model was carried out to examine the relationship between the numbers of students per doctor. The unit for analysis on this regression was the published studies that describe global surgery courses. Studies were included in the regression if they provided sufficient information to analyze the dependent variable and complete information on the covariates included in the final model.

The dependent variable was the ratio of the number of students from global surgery courses per 100,000 doctors in that country (students/100,000 doctors) in each country.

The independent variables included in the model were categorized as follows: course focus, course country, study design, income classification of the first author’s country (HIC or LMIC), income classification of the course country, and university access in the course country (rates of academic access to university in the interest course country).

Course focus was categorized as trauma, neurosurgery, other specialties, general surgery, NDA, and general and urology, with general surgery used as the reference category. Course country was entered as a categorical variable.

The countries included was Botswana, Canada, Dominican Republic, Ethiopia, Ghana, Haiti, Indonesia, Kenya, Mexico, Myanmar, Nigeria, Rwanda, South Korea, Tanzania, Uganda, United Kingdom, United States of America, and Zimbabwe, with Angola serving as the reference category.

The study design was categorized as cross-sectional study, descriptive study, mixed-methods study, non-randomized experimental study, randomized controlled trial, survey, and cohort study. Theoretical essays were not included in the regression analysis, because they did not provide objective quantitative data required to calculate the dependent variable. Cohort studies were used as the reference category for quantile regression.

A median quantile regression was chosen because it is less influenced by asymmetry in the data, non-normal distributions, and extreme observations than ordinary least-squares regression, given the relatively small sample size and the distribution of the dependent variable. Also, this approach estimates the central tendency of the outcome without assuming normal distribution.

## Results

We uploaded 2,534 articles on covidence, and 1,494 duplicates were automatically excluded. Then, 1,040 titles and abstracts were screened, of which 790 were excluded. Finally, 250 underwent full-text review. Of those undergoing full-text review, 46 did not disclose information about global surgery curriculum, 41 were surveys about interest in global surgery courses, 24 did not present full-text available and 20 were literature reviews. Therefore, they were not included in our study design methodology, leaving 119, which were included in the final analysis (Fig. 1).

**Figure 1:**
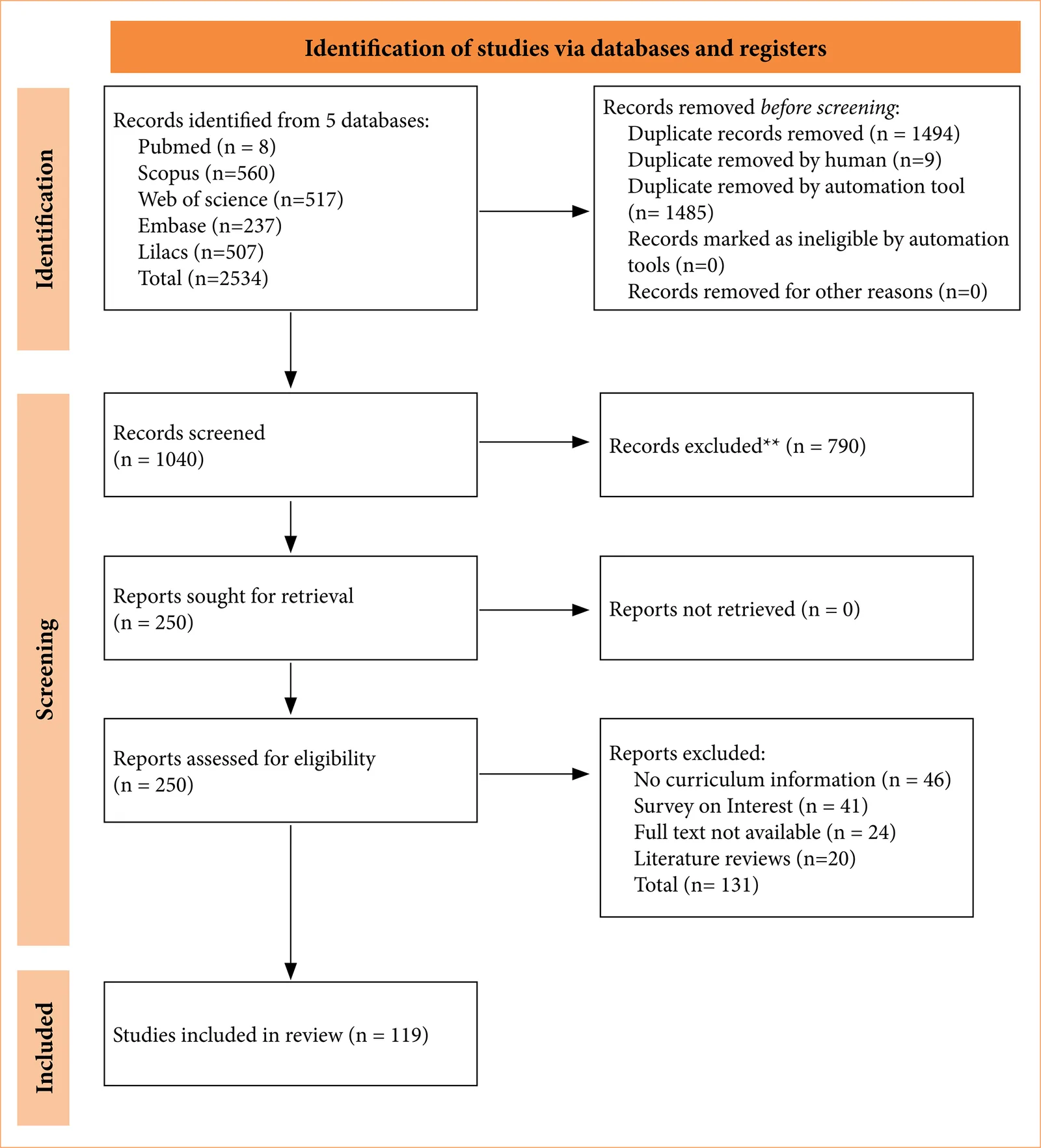
Preferred Reporting Items for Scoping Reviews and Meta-Analyses (PRISMA) flowchart.

Source: The PRISMA 2020 statement: an updated guideline for reporting systematic reviews^5^.

### Study characteristics

Regarding the study design, theoretical essays accounted for 18.49% (n = 22/119) of studies, cohort study represented 10.92% (n = 13/119), cross-sectional study were 7.56% (n = 9/119), and non-randomized experimental study represented 2.52% of studies (n = 3/119). The less representative study designs were mixed-methods study and randomized controlled trial, accounting 1.68% each (n = 2/119 each). The highest fraction of studies is represented by descriptive study (28.57%—n = 34/119) and surveys (28.57%—n = 34/119). Two studies did not have an explicit study design.

This study used a quantile regression model to examine the relationship between the number of students per doctor within each country and independent variables. The analysis included 42 published studies with complete data for the variables included in the final model. The model had a pseudo-R^2^ = 0.8906. The model was adjusted over two iterations to minimize the sum of weighted deviations—an initial weighted deviation of 159.86 and a final minimized value of 6.76. Collinearity led to the exclusion of one of the course’s variable focus categories (pediatric surgery) from the final model.

By study design, the surveys (0.13, 95% confidence interval [95%CI] -0.17–0.34, *p* = 0.371) and descriptive studies (0.23, 95%CI -0.19–0.47, *p* = 0.231) had a positive coefficient, but they were not statistically significant. The cross-sectional studies (-28.49, 95%CI -30.95 to -26.02, *p* < 0.001), mixed-methods studies (-22.45, 95%CI -25.32 to -19.58, *p* < 0.001), non-randomized experimental studies (-10.54, 95%CI -12.01 to -9.07, *p* < 0.001), and randomized controlled trials (-36.19, 95%CI -39.91 to -32.47, *p* < 0.001) indicated a significant association with lower numbers of global surgery students per doctors.

### Students relative to doctor per country

Course country was significantly associated with the dependent variable for Canada (β = 8.74, 95%CI 5.55–11.92; *p* < 0.001), Indonesia (β = -18.65, 95%CI -21.06 to -16.24; *p* < 0.001), Kenya (β = 11.64, 95%CI 9.36–13.92; *p* < 0.001), Myanmar (β = 37.21, 95%CI 32.84–41.57; *p* < 0.001), Nigeria (β = -10.72, 95%CI -14.59 to -6.84; *p* < 0.001), South Korea (β = -38.03, 95%CI -41.22 to -34.84; *p* < 0.001), and the United States of America (β = 30.02, 95%CI 25.81–34.24; *p* < 0.001), compared with the reference country (Angola). Other country coefficients were not statistically significant (Fig. 2).

**Figure 2:**
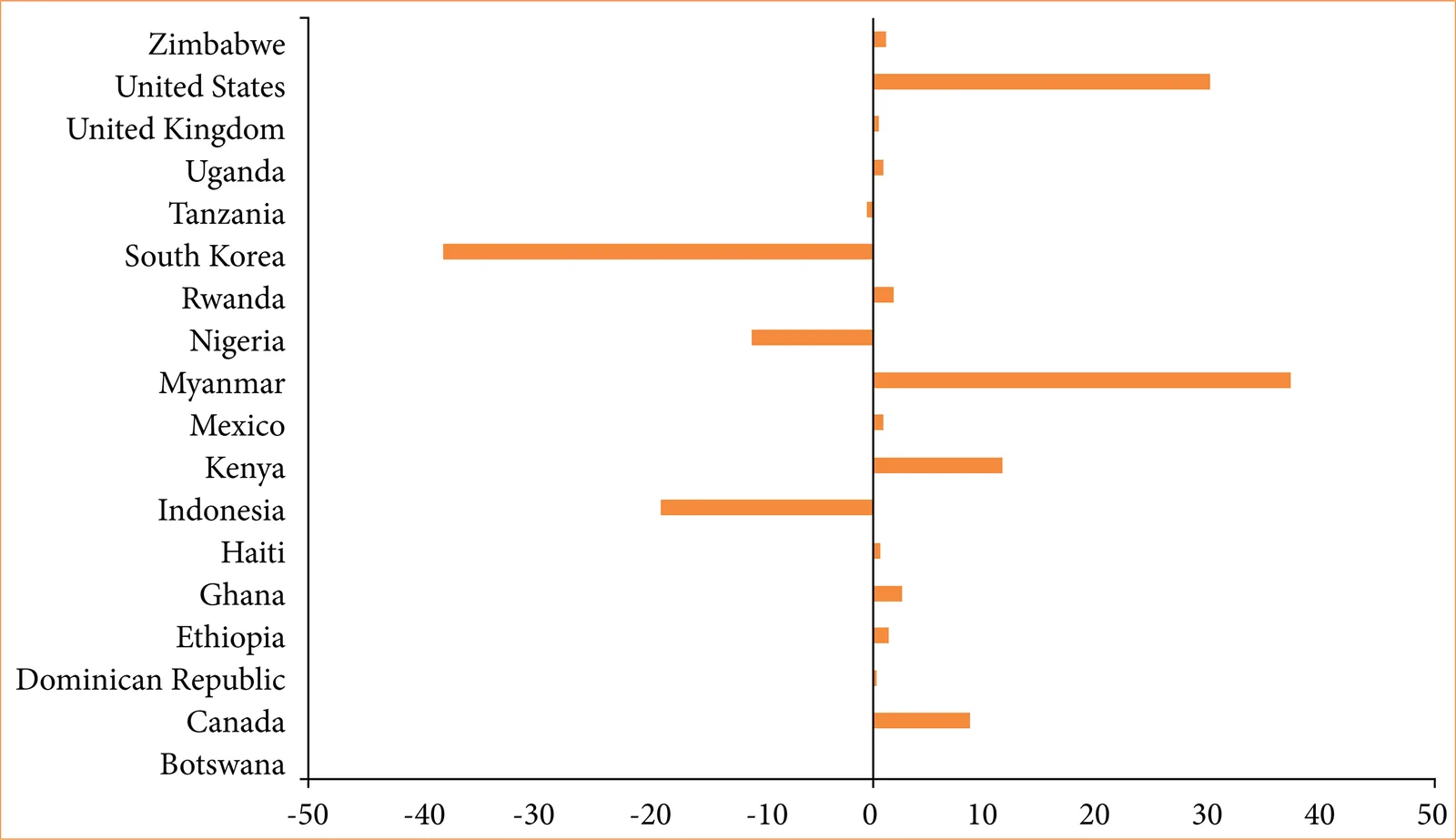
Coefficients from quantile regression model on log-sign transformed variables. This bar plot depicts the countries’ coefficients from a quantile regression analysis, with the response variable transformed using the log-sign method. The x-axis represents the magnitude and direction of the coefficients, with positive values to the right and negative values to the left.

Source: Elaborated by the authors.

### Courses by health specialties

For course focus, the NDA had the largest negative coefficient among course focuses (-33.51, 95%CI -37.71 to -29.30, *p* < 0.001), suggesting that courses without a defined specialty focus had a significant negative association on the dependent variable.

### Course by country-income level

The income level of the authors’ country had a negative association between the income level of the author’s country and the number of global surgery students relative to the physician workforce (-34.82, 95%CI -40.75 to -28.89, *p* < 0.001). On the other hand, this shows the correlation between courses offered in HICs and a higher ratio of global surgery students per doctors compared to courses offered in LMICs.

In contrast, the income level of the course country had a significantly positive coefficient (29.35, 95%CI 25.55–33.14, *p* < 0.001), suggesting that courses offered in HIC are positively associated with the dependent variable.

The university access in the course country had the most significant negative coefficient in the model (-104.83, 95%CI -116.49 to -93.17, *p* < 0.001), suggesting that limited university access in the course country is associated with a substantial negative association on the dependent variable.

The constant in the model was 68.65 (95%CI 56.27–81.03, *p* < 0.001), which is the baseline level of the dependent variable when all other factors are held constant.

### Course specificities

Almost half of the courses addressed general medical education (46.28%, n = 56/119). General surgery corresponded to 13.22% (n = 16/119) of the courses, followed by neurosurgery and pediatric surgery, both with 7.44% (n = 9/119), and trauma, with 6.61% (n = 8/119). Other specialties included were urology, plastic surgery, and orthopedic surgery, with 2.48% (n = 3/119) each. Only 1.65% of courses addressed anesthesiology or obstetrician-gynecology (n = 2/119, each). Other courses focused on hand surgery, maxillofacial surgery, military, minimally invasive surgery, and rural surgery, with 0.83% (n = 1/119) each, and 4.13% (n = 5/119) of the courses did not mention any specialty.

Among the papers reviewed, 36.13% (n = 43/119) did not report whether the course was practical, theoretical, or both. Only 7.56% (n = 9/119) of the courses were completely practical, 23.53% (n = 28/119) were all theoretical, and 32.77% (n = 39/119) had both theory and practice on their syllabi. Regarding the modality of the course, almost half of the papers reported in-person courses (42.02%—n = 50/119), followed by 18.49% (n = 22/119) virtual, and 6.72% (n = 8/119) hybrid. In contrast, 32.77% (n = 39/119) did not mention the course modality directly. Among the courses with practical content, 4.17% (n = 5/119) of the total courses analyzed had both simulation and patient-based trainings, 15.83% (n = 19/119) had only patient-based training, 14.17% (n = 17/119) had only simulation training, and 0.83% (n = 1/119) had cadaveric models. Also, 5% (n = 6/119) did not specify whether simulation or patient-based training was included. Additionally, 15.13% (n = 18/119) of the courses had research-oriented teaching, while the remaining courses did not involve or did not mention this topic.

Out of the 119 papers analyzed, 32 had more than one target group of students in their courses. Because of this, the total number of student groups considered was 151 (119+32). Residents (27.15%—n = 41/151) and medical school graduates (22.52%—n = 34/151) were the predominant audience across the courses followed by medical students (15.23%—n = 23/151) and attendings (10.60%—n = 16/151). Others varied between interns (5.96%—n = 9/151), fellows (3.97%—n = 6/151), other health care professionals (3.31%—n = 5/151). Some studies did not specify the target audience (9.93%—n = 15/151). While we primarily attempted to differentiate medical students by their year in school, 92.5% (n = 111/119) of the courses did not specify the year of study for attendees. Of those that mentioned the year of study, only 1.67% (n = 2/119) of the courses included students in their final years, and 0.83% (n = 1/119) included students from other years.

### Countries involved in the global surgery courses

Most of the papers were first authored by providers from the United States of America, with 68.91% of the articles (n = 82/119), followed by Canada, with 4.2% of the articles (n = 5/119). South Africa and Kenya represented 3.36% (n = 4/119 each), while Sweden, Nigeria, and Australia had 1.68% (n = 2/119 each). Regarding student demographics, 62.18% (n = 74/119) of the studies did not report the number of students participating in the courses. This lack of information makes it difficult to accurately compare the number of students from LMICs or HICs who benefited from the course.

Considering all the papers selected (n = 119), 50.42% (n = 60/119) of the reported courses took place in LMICs, while 26.05% (n = 31/119) took place in HICs, and 4.20% (n = 5/119) were offered through partnerships involving both LMICs and HICs. In 19.33% (n = 23/119) of the studies, the countries benefiting from the courses were not explicitly reported. The locations in HICs included Australia, the United Kingdom, Canada, and the United States of America. On the other hand, the locations in LMICs included Malawi, Mexico, Costa Rica, Angola, Guinea-Conakry, Ghana, Cameroon, the Republic of Congo, Kenya, Ethiopia, Madagascar, Belize, Botswana, Burundi, Mozambique, Namibia, Rwanda, South Sudan, Sudan, Tanzania, Uganda, Zambia, and Zimbabwe, the Caribbean, Guatemala, India, Indonesia, Kenya, Nigeria, South Africa, Malawi, Pakistan, Philippines, Sierra Leone, South Korea, Kampala, Mbarara, Gulu, the Dominican Republic, Myanmar, and Haiti.

By studying the publications per year, we can see that the number of global surgery courses per year are increasing and most courses are offered in LMICs (Figs. 3 and 4).

**Figure 3:**
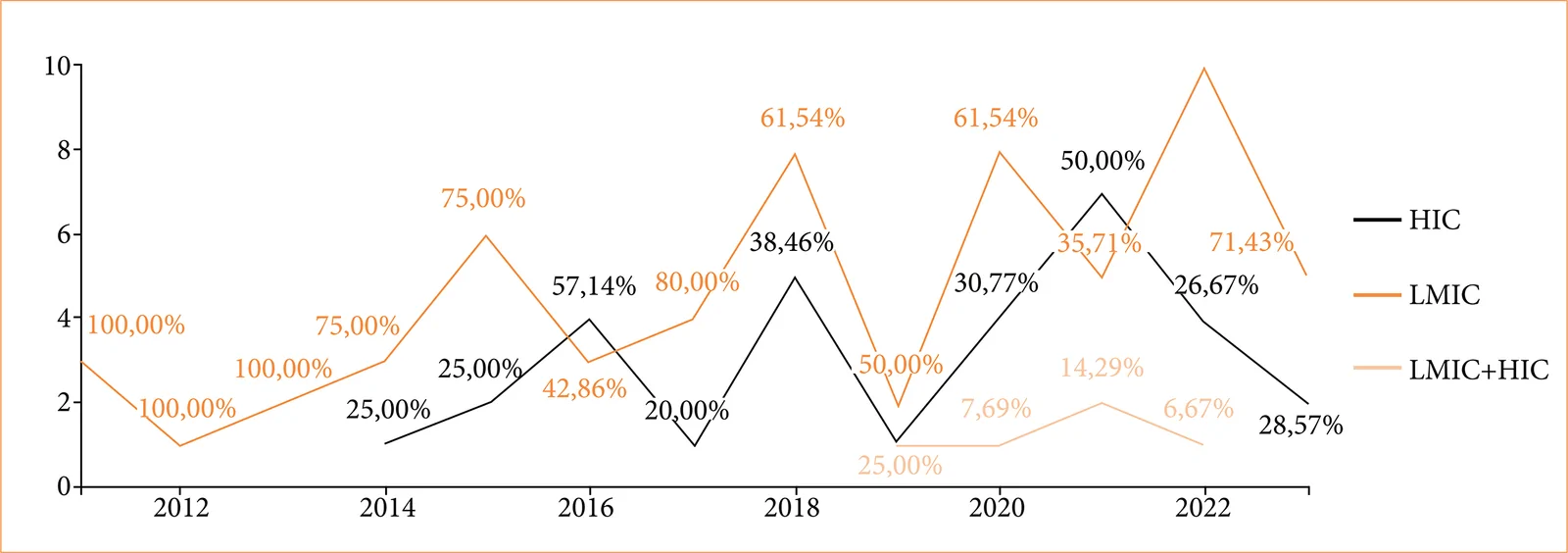
Number of courses in each type of location, per year.

HIC: high-income country; LMIC: low- and middle-income country. Source: Elaborated by the authors.

**Figure 4:**
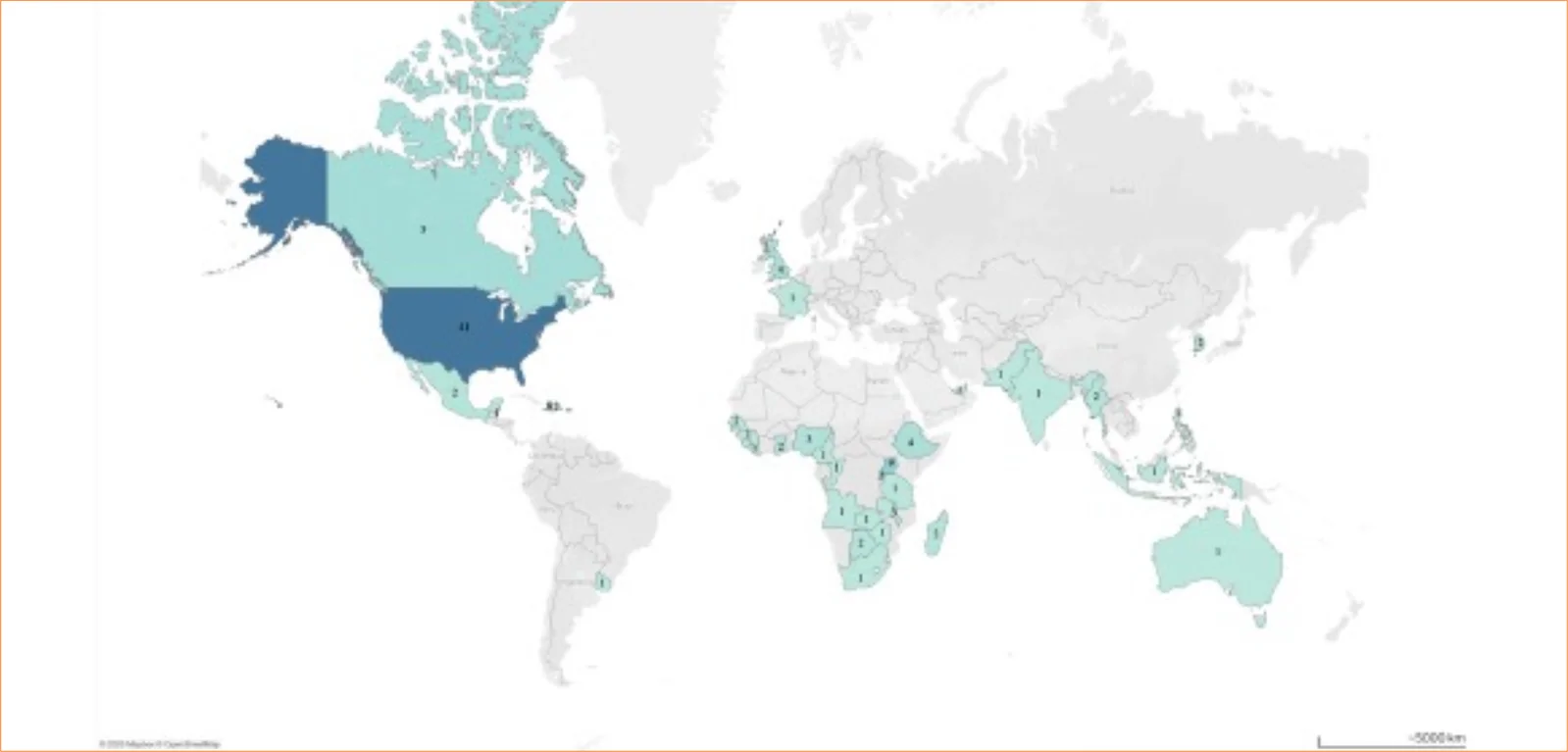
Map of the prevalence of courses by country. The countries are shaded according to the number of courses in each country.

Source: Elaborated by the authors.

## Discussion

### Global surgery as theoretic content

Even before the formal inception of global surgery in 2015, a variety of initiatives have sought to advance education in the field. Knowledge sharing has largely taken the form of webinars, workshops, and short-term courses that have helped raise awareness and connect a growing community of professionals. Online initiatives, including The Surg Hub by the Global Surgery Foundation, UNITAR, University of Oxford’s Global Surgery short course, and a range of collaborating partners, have broadened access to education by offering digital courses that connect learners and faculty across different regions^6^. Fellowship opportunities (examples on Table 1) such as Harvard’s Program in Global Surgery and Social Change, the Global Surgery Foundation fellowship, the Operation Smile Global Surgery Advocacy Fellowship, the Jean-Martin Laberge Fellowship in Global Pediatric Surgery, Center for Health Equity in Surgery and Anesthesia (CHESA) at University of California, San Francisco (UCSF), University of California in Davis, and Baylor College of Medicine have provided training in policy, advocacy, and research.

**Table 1:** Links to academic opportunities in global surgery.

Institution	Website
Baylor College of Medicine	https://www.bcm.edu/departments/surgery/education/training-programs/general-surgery-residency/global-surgery-track
McGill University	https://www.mcgill.ca/pediatricsurgery/education/fellowships/jean-martin-laberge-fellowship-global-pediatric-surgery
University of California in Davis Health	https://health.ucdavis.edu/surgery/global-surgery/
Harvard Medical School	https://www.pgssc.org/
Operation Smile	https://www.operationsmile.org/global-health-equity/policy-advocacy/global-surgery-advocacy-fellows/
The Global Surgery Foundation.	https://www.globalsurgeryfoundation.org/
McMaster University	https://gs.mcmaster.ca/program/global-surgery/
University of California, San Francisco	https://globalsurgery.org/
University of CapeTown	https://health.uct.ac.za/global-surgery/education
Stellenbosch University	https://www.su.ac.za/en
University of Global Health Equity	https://ughe.org/centre-for-equity-cegs-in-global-surgery-fellowship/
College of Surgeons of East, Central and Southern Africa	https://www.cosecsa.org/

Source: Elaborated by the authors.

Despite these incredible efforts, global surgery is not established as a formal academic discipline yet, particularly in LMICs. Universities worldwide should move beyond temporary or extracurricular activities and create dedicated programs or departments that ensure continuity and structure. Integrating global surgery into the core curriculum would allow it to become a sustained part of medical training^7-9^.

Our findings echo previous studies that emphasize the limited availability of structured education in global surgery and the ongoing challenges of developing courses that are standardized, accessible, and adapted to local healthcare needs^3,8^. A recent modified Delphi process involving experts from 22 countries developed a consensus framework for global surgery competencies, emphasizing the need to address both international and local contexts, as well as the incorporation of cultural, gender, and community-specific factors^7^. To ensure inclusivity and alignment with global priorities, the study proposes a curriculum that expands its focus on equity.

Great examples of institutions leading the way in expanding global surgery curricula include those that address not only the clinical aspects of care but also the social, political, and environmental determinants impacting surgical access and outcomes. In 2015, the University of British Columbia launched its Master of Global Surgical Care program, a fully online, two-year course designed to equip professionals with the skills needed to address surgical challenges in low-resource settings^7^. The program offers courses such as Surgical Care in International Health and Surgical Care in Humanitarian Disaster Response, along with a field practicum to provide hands-on experience in underserved areas. Building on these efforts, Duke University, in partnership with the University of Global Health Equity in Rwanda, developed the Global Surgical Care course^10^. This hybrid program examines barriers to surgical access in many LMICs and explores strategies for improving surgical care within these health systems.

Other universities have formal master’s programs in global surgery, such as Oxford, McGill and King’s College London. More recently, McMaster University introduced its graduate diploma in global surgery, a program aiming to address contemporary issues, such as ethics, gender equality, planetary health, and humanitarian crises, with lectures delivered by faculty from LMICs and various disciplines (Table 1). The course also covers the unique challenges faced by distinct populations in global surgical care, including indigenous/first nations communities and refugees.

Despite these significant contributions, as observed in our results, global surgery education is still predominantly delivered in HICs, often by institutions with limited exposure to LMIC settings. Notable examples include the University of Global Health Equity in Rwanda with its Center for Equity in Global Surgery, along with the University of Cape Town and Stellenbosch University in South Africa, all contributing to local capacity building through global surgery programs (Table 1). This highlights the need for further expansion of locally driven, contextually relevant programs in LMICs universities to ensure that global surgery education addresses the specific challenges faced by these regions.

### Global surgery applied to training courses

With a limited surgical workforce in many settings, clinical training is essential. However, developing courses in resource-limited environments, especially for specialized fields that require additional resources, present further complexities^11^. Challenges in global surgery include language barriers, country-specific epidemiology, funding, and the availability of materials and resources. These factors emphasize the need to contextualize the curriculum to each country’s unique circumstances. Surgical education also includes non-technical skills, such as communication, teamwork, and decision-making, which remain limited or unknown in LMICs and should be further developed^12,13^.

Although general medical training, including general surgery, remains essential, we observed that 42% of programs focus exclusively on general medical education. While providing basic knowledge is valuable, there is a growing need for specialized training to expand surgical capacity in specific areas to ensure that specialized skills are also cultivated, particularly in LMICs where workforce shortages are most acute. Specialty courses should therefore be increasingly explored and developed to address the diverse surgical needs in resource-limited settings. Initiatives such as the anesthesia fellowship at the University of Global Health Equity, the Global Trauma Resuscitation in Kids program for pediatric trauma, and College of Surgeons of East, Central and Southern Africa (COSECSA)’s fellowships (Table 1) illustrate how targeted specialty education can strengthen surgical care^12,^ ^14^.

Another finding was that courses were often limited to theoretical content, with few providing meaningful practical training models. In our review, over one-third did not specify their format, only a small fraction (7.6%) was fully practical, and less than one-third combined theory and practice. Similarly, while most programs were reported as in-person, few offered virtual or hybrid options, and many did not describe their modality. Among those with hands-on content, only a small number incorporated simulation training. This reflects a missed opportunity to integrate innovative teaching methods into global surgery curricula. Low-cost simulations and virtual reality have already shown promise as practical training tools in resource-limited settings^15-17^. In Uganda, a United Kingdom–Uganda partnership developed a VR training module using cadaveric footage and headsets for obstetrics, trauma, and general surgery training, while in India, the Christian Medical College Ludhiana and McGill University launched a VR program for pediatric trauma^18,19^. Affordable three-dimensional-printed simulators have been validated, including laparoscopic trainers, modular pediatric models, and low-cost liver and otolaryngology simulators^20,21^. Besides skepticism, affordable technologies can fill critical gaps in practical training and strengthen surgical education where traditional resources are scarce.

Another important consideration is the audience to whom global surgery education is directed. We observed that clinical training opportunities are often limited to medical and surgical trainees, with only 3% of studies identified in our results, extending to other health care professionals. Yet, professions such as nursing, midwifery, and anesthesia providers are indispensable within the health system, particularly in LMICs where task-sharing is essential to expanding surgical access. Strengthening training opportunities for these professionals is crucial to ensure that surgical care is delivered safely, effectively, and equitably across diverse health care teams.

Geographically, we also observed that clinical training in global surgery is predominantly planned and executed by institutions in HICs, even when these programs are delivered in LMICs. While there has been an effort to bridge these gaps, the predominant involvement of HICs in program development may lead to misaligned priorities. Highlighting the importance of fully engaging LMIC authors in developing scientific outcomes is essential^22^. For instance, curricula developed in HICs may not sufficiently address the specific epidemiological concerns or resource constraints of LMICs, leading to less practical or applicable training. These differences likely reflect the varied objectives of coaching programs.

Moreover, we observed various levels of involvement, ranging from fundraising to in-person overseas work. However, there is limited information on how HIC trainee visits benefit local populations or how these short-term visits impact the training of LMIC trainees^23^. To address these disparities, global surgery programs must prioritize leadership and input from local stakeholders in LMICs to ensure training is relevant, effective, and sustainable. Partnerships between HICs and LMICs should emphasize locally tailored content that develops both technical and non-technical skills within existing health systems. There is also a need to expand programs in LMICs and led by LMICs. Initiatives such as the University of Global Health Equity in Rwanda, which hosts the Center for Equity in Global Surgery, and COSECSA’s regional training model across 14 countries, are clear examples of the strength of local leadership and the power to create durable and context-specific frameworks (Table 1). These efforts show the potential of LMIC institutions to shape global surgery education, underscoring the importance of moving beyond HIC-dominated models to build more equitable and effective systems.

This study is not exempt from limitations. First, most of the examined articles were observational studies, which could lead to study design drawbacks, including biases in recollection and selection. Additionally, the outcomes of global surgery courses in specific LMICs were not differentiated, limiting our understanding of contextual effectiveness. Studies with larger sample sizes were also scarce, likely due to the emerging nature of formal academic courses in this field, which may contribute to selection bias. Furthermore, we did not collect data on the students’ nationality or the specific locations of the courses separately. Finally, another limitation of our study is the limited number of global surgery courses that have published their curricula in scientific journals. Consequently, it is important to emphasize that our statistical results reflect only courses whose curricula have undergone scientific publication. Future studies on global surgery courses would benefit from reporting these variables to enable more precise analysis.

## Conclusion

While global surgery has grown rapidly since its formal inception in 2015, its educational framework remains underdeveloped. Our study revealed that, despite being available, practical surgical courses lack standardized curricula that address cultural and socioeconomic factors essential for global applicability. Additionally, although these courses are frequently held in LMICs, most of the research is led by authors affiliated with HIC institutions, indicating a need for more local perspectives.

Beyond practical and technical training, the integration of global surgery as an educational discipline remains underdeveloped. There is a need to rethink global surgery education through innovation, diversity, inclusivity, sustainability, collaboration, and leadership. Introducing global surgery concepts at the undergraduate level could foster early awareness of social diversity and health care disparities, laying the foundation for a more inclusive and contextually relevant approach to surgical training. This study offers insights for institutions seeking to develop global surgery programs by identifying current strengths and areas for improvement, and we encourage stakeholders to address these gaps to better reflect the diverse needs of global communities.

## Data Availability

All dataset are presented in the article.
